# Cyclodextrin-Based Displacement Strategy of Sterigmatocystin from Serum Albumin as a Novel Approach for Acute Poisoning Detoxification

**DOI:** 10.3390/ijms24054485

**Published:** 2023-02-24

**Authors:** Daniela Jakšić, Maja Šegvić Klarić, Hrvoje Rimac, Robert Kerep, Ivo Piantanida

**Affiliations:** 1 Department of Microbiology, Faculty of Pharmacy and Biochemistry, University of Zagreb, 10000 Zagreb, Croatia; 2 Department of Medicinal Chemistry, Faculty of Pharmacy and Biochemistry, University of Zagreb, 10000 Zagreb, Croatia; 3 Department of General and Inorganic Chemistry, Faculty of Pharmacy and Biochemistry, University of Zagreb, 10000 Zagreb, Croatia; 4 Division of Organic Chemistry and Biochemistry, Ruđer Bošković Institute, Bijenička Cesta 54, 10000 Zagreb, Croatia

**Keywords:** mycotoxin sterigmatocystin, cyclodextrin, competitive binding, fluorescence, circular dichroism

## Abstract

This study demonstrates that sterigmatocystin (STC) interacts non-covalently with various cyclodextrins (CDs), showing the highest binding affinity for sugammadex (a γ-CD derivative) and γ-CD, and an almost order of magnitude lower affinity for β-CD. This difference in affinity was studied using molecular modelling and fluorescence spectroscopy, which demonstrated a better insertion of STC into larger CDs. In parallel, we showed that STC binds to human serum albumin (HSA) (a blood protein known for its role as a transporter of small molecules) with an almost two order of magnitude lower affinity compared to sugammadex and γ-CD. Competitive fluorescence experiments clearly demonstrated an efficient displacement of STC from the STC–HSA complex by cyclodextrins. These results are a proof-of-concept that CDs can be used to complex STC and related mycotoxins. Similarly, as sugammadex extracts neuromuscular relaxants (e.g., rocuronium and vecuronium) from blood and blocks their bioactivity, it could also be used as first aid upon acute intoxication to encapsulate a larger part of the STC mycotoxin from serum albumin.

## 1. Introduction

**Scheme 1:**
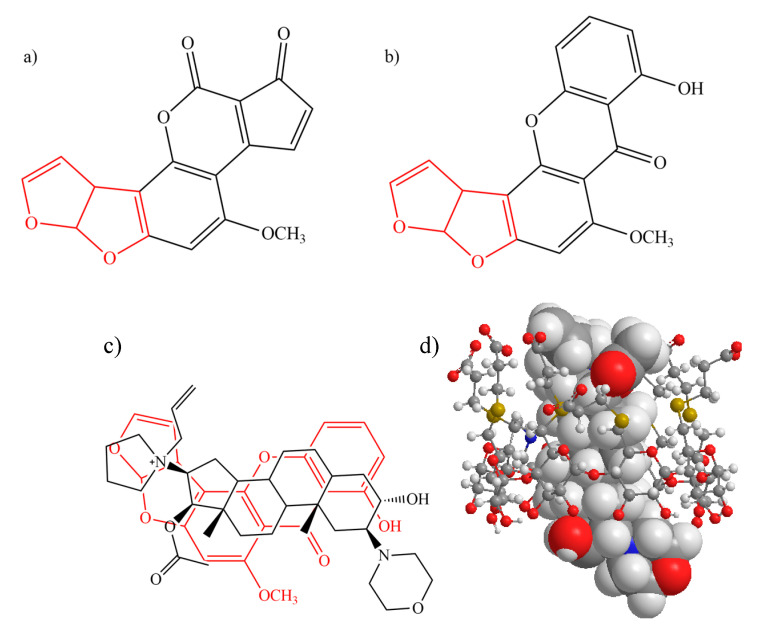
Structures of AFB_1_ (**a**) and STC (**b**); (**c**) structure similarity of studied STC (in red) and neuromuscular blocking agent rocuronium (in black) (drawn in ChemDraw Ultra 12.0); and (**d**) rocuronium in a complex with cyclodextrin derivative sugammadex [20].

Human serum albumin (HSA) is the most abundant serum protein. Along with its numerous physiological functions, which include the regulation of oncotic pressure and transport of fatty acids, hormones, bile salts, amino acids, metals, and toxic metabolites, it also participates in the transport of drugs and xenobiotics to their target organs or tissue. The interactions of drugs and xenobiotics with HSA are important to monitor because of their role in distribution, metabolism, and elimination of drugs and xenobiotics, as well as in the assessment of their desired and undesired effects [1]. Similarly, compounds with the ability to displace other drugs/xenobiotics from HSA may be interesting agents in action modulation of the target drug/xenobiotic. In this research, we focused on mycotoxins, small molecules with documented toxicity produced as the secondary metabolites by fungi from various genera. As people are exposed to mycotoxins through various organic and inorganic substrates via ingestion or inhalation, interactions between mycotoxins and HSA are well documented [2]. Aflatoxin B_1_ (AFB_1_), one of the most toxic naturally occurring substances and a group 1 carcinogen [3], is the best-known and the most thoroughly studied mycotoxin produced by Aspergilli from the section *Flavi*. In this research, we focused on sterigmatocystin (STC), a less thoroughly investigated derivative of AFB_1_, since people may be more exposed to STC as one of the most common indoor mycotoxins [4,5,6]. Research conducted between the 1970s and the early 1990s has revealed that STC toxicity involves the formation of a reactive epoxy group which covalently binds to a DNA molecule by creating a STC-N_7_-guanine adduct [7,8]. However, STC toxicity is claimed to be three to ten times lower than the toxicity of AFB_1_ [9]. Although STC and AFB_1_ share the bis-furane structural moiety (Scheme 1a,b), STC is characterized by a larger aromatic and hydrophobic surface (having one additional condensed ring) and thus the aromatic and hydrophobic non-covalent interactions may be significant in its interactions with various biomacromolecules. Indeed, we have recently shown that, in contrast to AFB_1_, STC forms very stable aggregates in aqueous solutions, yielding a specific and strong induced circular dichroism (ICD) signal close to the visible range (ICD band at 350 nm) [10]. We used this ICD signal to study non-covalent STC interactions with DNA, which we proposed as an intermediate stage to covalent DNA damage, a well-known mode of STC toxicity [7,8]. However, body uptake of STC or AFB_1_ upon ingestion, inhalation, or through the skin requires transport of these mycotoxins by blood, commonly involving serum albumin, the most abundant blood protein and a well-known transporter of small molecules [11]. Following ingestion of food contaminated with AFB_1_, HSA may form covalent adducts with up to 2.3% of absorbed AFB_1_ [12]. A recent study showed that STC has a moderate affinity for HSA (log*K_SV_* 3.98 ± 0.06 M^−1^, obtained by fluorescence spectroscopy) and, based on molecular modelling, the heme binding site was suggested as the most probable STC binding site [13]. Consequently, a strategy aiming toward the first aid detoxification upon acute exposure to mycotoxins could involve competitive extraction of toxins from HSA to form an inert complex, with the latter being safely excreted from the body afterwards. 

Cyclodextrins are particularly suitable for eliminating toxic substances in vivo because their lipophilic cavity enables formation of complexes with lipophilic small molecules, while at the same time they are perfectly soluble in physiological fluids due to their polyhydroxy hydrophilic coat. This has been adopted in the pharmaceutical industry and cyclodextrins are widely used as solubilizing agents as well as antidotes. Sugammadex, a registered drug prescribed for the reversal of block anaesthesia is a great example of an antidote [14]. Sugammadex is an anion derivative of cyclodextrin-γ (γ-CD), binds rocuronium specifically with a very high affinity (Scheme 1d; *K*_a_ = 1.03 × 10^7^ M^−1^) [15], and is administered intravenously to extract rocuronium and stop its biological effects within several minutes of administration. In general, plenty of data have demonstrated cyclodextrins’ capacity to bind different small molecules [16,17]. For instance, mycotoxin AFB_1_ mostly forms an inclusion complex in cyclodextrin’s hydrophobic interior, resulting in enhanced AFB_1_ fluorescence [18]. Molecular modelling data indicate that insertion of the bis-furane AFB_1_ group into β-CD is supported by hydrogen bonds formed between the carbonyl AFB_1_ group and secondary hydroxyl β-CD groups [19]. Regarding the planar and hydrophobic condensed cyclic core, there is a remarkable structural similarity between rocuronium and STC (Scheme 1c,d), which inspired this research. Thus, the aim of this study was to assess the binding affinity of STC to sugammadex, as well as to closely related cyclodextrins (β-CD and γ-CD), to determine the impact of the CD-ring size and substitution on STC complexation.

Previous research has shown that cyclodextrins are useful additives in improving the fluorescent properties of mycotoxins [21], and because of their binding capacity they may be useful in removing mycotoxins from food and feed [22]. Several studies have shown that cyclodextrins can be useful in extracting mycotoxins from aqueous solutions and beverages such as beer, wine, and juices [23,24,25], but interactions of STC and CDs have been scarcely studied. One type of a fluorescent probe, based on carbon nitride nanoparticles, was modified with β-CD for the purpose of optimizing the method for STC detection in cereals [26]. More recently, it was shown that a β-CD polymer binds up to 80% of STC (2 μM) in buffered acidic to neutral aqueous solutions [22].

In this paper, the non-covalent interactions between STC and β-CD, γ-CD, and sugammadex were explored and compared to interactions between STC and HSA. Competitive fluorescence experiments were conducted to demonstrate displacement of STC from the STC-HSA complex by cyclodextrins, which was also confirmed by molecular dynamics simulations. The affinity was calculated as log*K*_s_ from experimental data obtained by UV/Vis and CD spectroscopic methods, as well as isothermal titration calorimetry (ITC). Furthermore, STC displacement from the STC-HSA complex was examined by fluorometric titration of the pre-formed STC-HSA complex with sugammadex, β-CD, and γ-CD.

## 2. Results and Discussion

### 2.1. Study of STC Interactions with γ-CD, Sugammadex, β-CD, and HSA by Spectrophotometric Methods

We have previously shown that in aqueous solutions, STC forms very stable non-covalent aggregates, dissociating only at submicromolar concentrations [10]. Thus, STC binding to cyclodextrins or serum albumin in aqueous solutions would actually be a competitive process between STC aggregation and STC–host complexes. Fortunately, we discovered that STC aggregates are characterised by a specific and strong signal in circular dichroism (ICD band at 350 nm, with STC alone not having any CD spectrum) [10], which allows us to indirectly study STC–host complexation.

**Figure 1:**
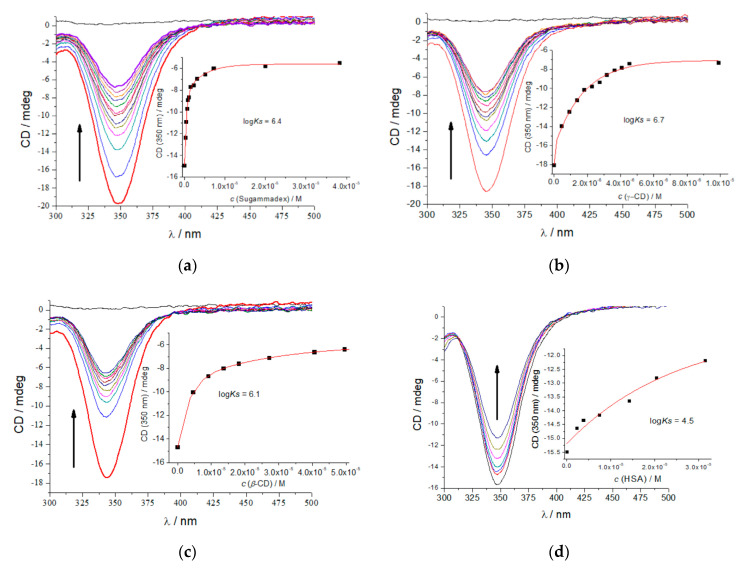
Changes in the STC aggregate (*c*(STC) = 2 × 10^−6^ M) CD spectrum upon addition of sugammadex (**a**), γ-CD (**b**), β-CD (**c**), and HSA (**d**). Each titration point is shown in different color and the direction of decrease in CD signal is indicated by an arrow. Inset: ICD band changes at *λ* = 350 nm fitted to the 1:1 stoichiometry complex (▬). Performed at pH 7.0 with Na-cacodylate buffer, *I* = 0.05 M.

Titration of STC aggregates with γ-CD, β-CD, and sugammadex resulted in a strong decrease in the ICD signal intensity at 350 nm (Figure 1a–c), whereas addition of HSA (Figure 1d) yielded a less pronounced ICD band decrease, significantly at an order of magnitude higher concentration in comparison to CDs. Fitting the titration data to the 1:1 stoichiometry complex model (STC:host) yielded apparent binding constants (Figure 1 insets and Table 1), which are attributed to STC aggregate dissociation caused by STC (single molecule) binding to the host (cyclodextrin or HSA).

**Figure 2:**
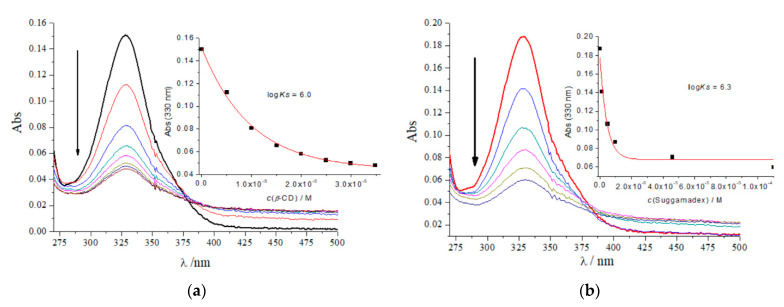
Changes in STC UV spectra (*c* = 1.8 × 10^−5^ M) upon titration with β-CD (**a**) and sugammadex (**b**). Each titration point is shown in different color and an the direction of decrease in UV signal is indicated by an arrow. Inset: absorbance changes at *λ* = 330 nm fitted to the 1:1 stoichiometry complex (▬). Performed at pH 7.0 with Na-cacodylate buffer, *I* = 0.05 M.

The results of CD experiments strongly support binding of STC to cyclodextrins and HSA; however, they should be verified by an independent method. It could be expected that a switch from an STC aggregate to an STC–host complex would have an impact on the STC UV/Vis spectrum. For that reason, we performed UV/Vis titrations, which showed a strong hypochromic effect upon addition of any CD (Figure 2 and Supp. Info. Figure S1), and fitting titration data to 1:1 stoichiometry complexes gave binding constants very similar to those determined by CD titrations (Table 1). An analogous STC titration with HSA was harder to perform due to HSA absorbance up to 300 nm; however, at low HSA concentrations, the hypochromic effect on the STC UV/Vis spectrum was visible (Supp. Info. Figure S1), supporting the STC–HSA complex formation.

So far, we have studied interactions of STC aggregated in an aqueous solution with various hosts, thus monitoring STC deaggregation as a consequence of binding to a host. Now, with the aim to directly monitor a single STC molecule binding to a host, we presumed that upon addition of a small aliquot of STC stock solution in acetonitrile (in which STC is not aggregated [10]) to a aqueous solution of a host macromolecule, STC will not have time to aggregate prior to forming an inclusion complex with a host. 

**Figure 3:**
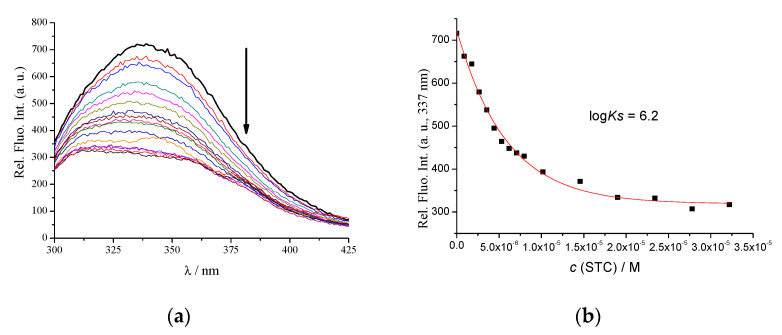
(**a**) Changes in HSA fluorescence emission spectra (*c* = 1 × 10^−6^ M, *λ*_exc_ = 280 nm) upon titration with STC (*c_stock_* = 9 × 10^−3^ M, in MeCN). (**b**) Emission changes at *λ* = 337 nm fitted to the 1:1 stoichiometry complex (▬). Each titration point is shown in different color and the decrease in fluorescence emission is indicated by an arrow. Performed at pH 7.0 with Na-cacodylate buffer, *I* = 0.05 M.

HSA is intrinsically fluorescent and binding of a small molecule to any of its binding sites causes a change in HSA fluorescence emission [1]. Conveniently, STC does not show fluorescence under the experimental conditions and the HSA excitation wavelength (*λ*_exc_ = 280 nm) coincides with negligible absorbance in the STC UV spectrum, thus avoiding the inner filter effect [27]. Addition of STC yielded a strong decrease in the HSA emission (Figure 3a) and fitting the titration data to the 1:1 stoichiometry complex (Figure 3b) allowed calculation of the real binding constant, defined by binding of a single STC molecule to the host.

Much lower values of the apparent binding constants obtained for the STC–HSA complex from CD and UV titrations (log*K*_s_ = 4.5–5.0) with respect to the binding constant obtained from fluorometric titration (log*K*_s_ = 6.2) can be attributed to the STC deaggregation process (present only in CD and UV titrations), which considerably competed with STC binding to HSA. Thus, addition of the STC acetonitrile solution to HSA allowed dominant binding of a single STC molecule to HSA, with no significant interference of STC aggregates. This also corroborates with a previously reported binding constant for the STC/HSA complex [13], which was performed with aggregated STC, thus yielding a lower apparent binding constant value (log*K_SV_* 3.98 ± 0.06 M^−1^). 

This fluorometric HSA titration with STC allowed us to plan competitive experiments, wherein an aqueous solution with a pre-formed STC–HSA complex would be titrated with various CDs, with the expected HSA emission increase attributed to the cyclodextrin-mediated STC extraction. It should be noted that in the absence of STC, the addition of any cyclodextrin did not influence HSA emission. Additionally, it is important to stress that the presumed STC extraction from HSA to cyclodextrin is not likely a result of a direct contact of HSA and cyclodextrin molecules (since they do not show any significant affinity to each other) but likely involves a portion of the free STC in solution, whereby these free STC molecules are prone to forming STC aggregates. 

**Figure 4:**
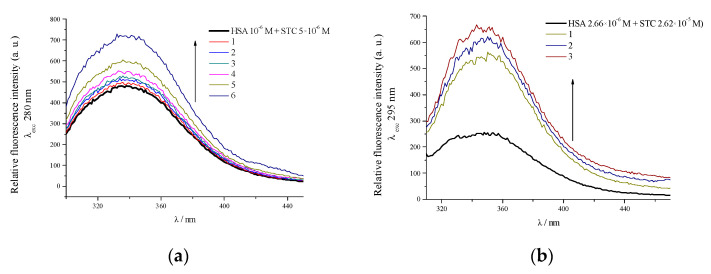

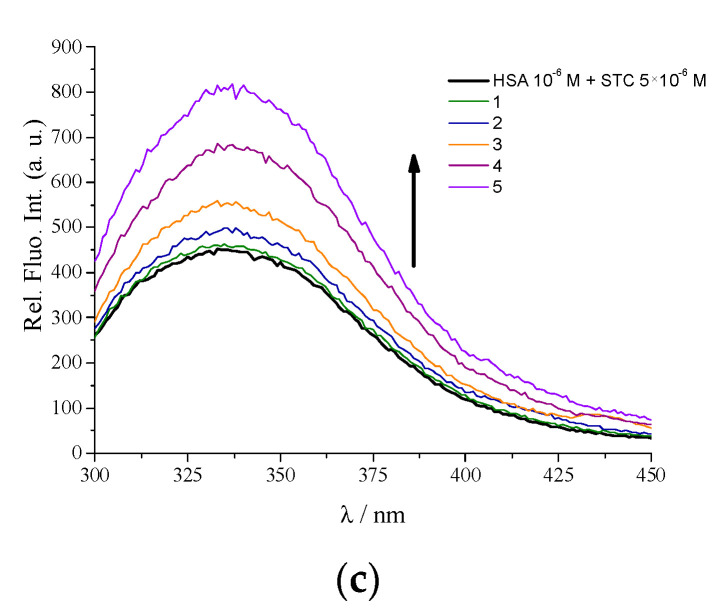
Changes in fluorescence spectra of the STC–HSA complex (*c*(HSA) = 1 × 10^−6^ M, *c*(STC) = 5 × 10^−6^ M) upon addition of (**a**) γ-CD, (**b**) sugammadex, and (**c**) β-CD. Each titration point is shown in different color and the direction of increase in fluorescence emission is indicated by an arrow. Performed at pH 7.0 with Na-cacodylate buffer, *I* = 0.05 M.

Although such a complex multicomponent system with several intertwined thermodynamic equilibria of different kinetics is not an ideal system for competitive experiments, the obtained results clearly demonstrate that addition of any cyclodextrin to the STC–HSA complex caused an increase in HSA fluorescence up to the maximum emission intensity of free HSA (Figure 4).

### 2.2. Study of STC Interaction with HSA and Sugammadex by Microcalorimetry

**Figure 5:**
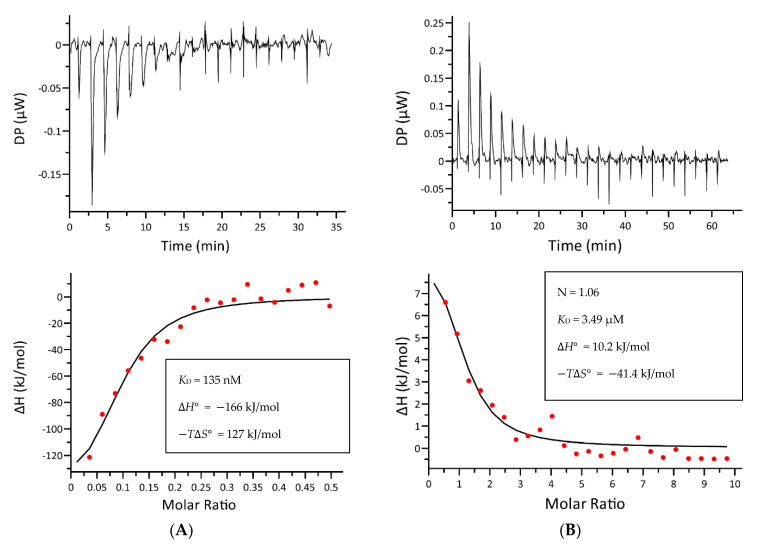
Microcalorimetric titration at 25 °C of STC (10^−5^ M in 200 µL sample cell). The top panel shows the heat in µW per injectant with subtracted baseline, the bottom panel shows the enthalpy change (kJ/mol) as a function of the STC/host molar ratio. After subtracting Δ*H*_dilution_ (control experiments with STC diluting itself), enthalpy data were fitted with a single binding site model. The insets show thermodynamic parameters (*K*_D,_ Δ*H*°, −*T*Δ*S*°. (−) calculated, ⦁ experimental. (**A**) Titration with HSA (2.5 × 10^−5^ M in 1.0 µL injections) and (**B**) titration with sugammadex (5 × 10^−4^ M in 1.0 µL injections).

The spectrophotometric experiments shown above revealed that STC can form strong non-covalent interactions with both HSA and cyclodextrins. To study these interactions by an independent method and to reveal more information on the thermodynamic profile, we decided to perform isothermal titration microcalorimetry (ITC) studies. However, STC has a low solubility in water and forms highly stable aggregates in aqueous solutions [10]; thus, experimental conditions applicable for ITC allowed only experiments with STC aggregate solution in a microcalorimeter cell. Titration of the prepared STC aggregate with any macromolecule solution (HSA and CD) would allow to monitor the process of STC deaggregation due to the binding of a single STC molecule inside a macromolecule. The obtained binding parameters (binding constant and stoichiometry of complex) are consequently only the apparent values, and are not directly comparable with the titration results (Figure 5), where changes in the intrinsic properties of HSA are proportional to the formation of a single molecule HSA–STC complex.

Indeed, titration of the STC aggregate with HSA (Figure 5A) revealed an exothermically driven binding interaction with a very low stoichiometry factor (N~0.1), suggesting the significant impact of the STC deaggregation process in the cumulative thermodynamics of the event. A correlation plot of the thermodynamic parameters reveals that an enthalpy–entropy compensation is in effect. The exothermic STC and HSA binding energetics (Figure 5A) are enthalpy driven by a combination of favourable (negative) enthalpic and unfavourable (negative) entropic contributions to the Gibbs free energy (Δ*G*°), which could be correlated to a combination of hydrophobic interactions and H-bonding interactions likely to happen within the HSA binding site. A negative value of Δ*G*° points to the spontaneity of the binding process.

In contrast to HSA, addition of sugammadex to the STC aggregate resulted in an endothermically driven binding interaction (Figure 5B). Again, a negative value of Δ*G*° points to the spontaneity of the binding process. Since the STC deaggregation process would likely be the same as in the HSA titration, the cumulative endothermic binding energetics in Figure 5B would imply a significant difference in the binding interactions of STC with HSA and sugammadex. A detailed analysis of the data shown in Figure 5B indicates dominating hydrophobic interactions, which is in accordance with a neutral ligand (STC) binding inside a highly hydrophobic host (sugammadex).

**Figure 6:**
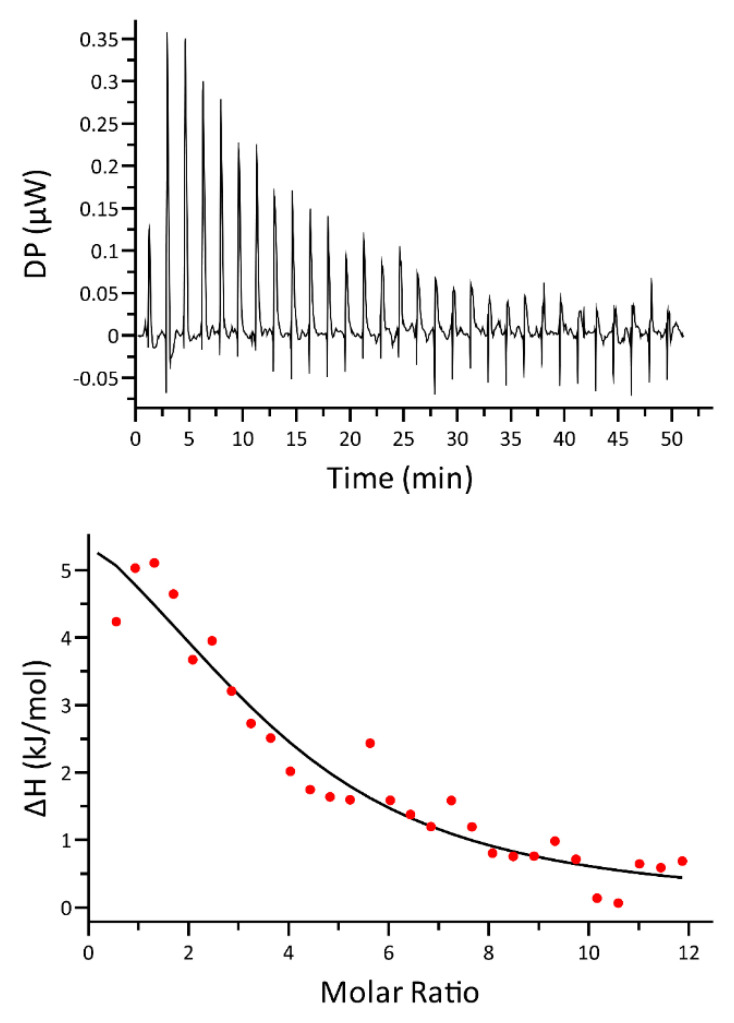
Microcalorimetric competitive titration of the STC–HSA complex with sugammadex (5 × 10^−4^ M in 1.0 µL injections) at 25 °C. The top panel shows the heat in µW per injectant with a subtracted baseline. The bottom panel shows the enthalpy change (kJ/mol) as a function of the sugammadex/complex molar ratio. After subtracting Δ *H*_dilution_ (control experiments with sugammadex and solvent dilution, as well as HSA–sugammadex heat interaction), enthalpy data were fitted to a single binding site model. (−) calculated, ⦁ experimental.

In order to compare and quantify STC aggregate interactions with HSA and sugammadex, we performed a competition experiment (Figure 6). Titration of the pre-formed STC–HSA complex inside the ITC sample cell with sugammadex displayed an endothermically driven binding interaction (Figure 6). The negative Gibbs free energy value indicates that the reaction occurred spontaneously and favourably. The binding reaction has a favourable entropy, *T*Δ*S*° = 35 kJ/mol, and an unfavourable enthalpy, Δ*H*° = 7.89 kJ/mol. The positive change in entropy is an indication of hydrophobic interactions and brings an additional contribution to the negative Gibbs free energy (Δ*G*° = –27.1 kJ/mol), while a small positive enthalpy change indicates few electrostatic interactions occurring. Considering that |Δ*H*°| < |*T*Δ*S*°|, the binding reaction occurring between the STC–HSA complex and sugammadex is driven mainly by changes in entropy. It should be stressed that titration of HSA alone with sugammadex did yield heat changes of approximately 4 kJ/mol, although complete HSA saturation was not achieved (Supp. Info. Figure S3).

Summarising the results of all titrations (Table 1), STC, although it is aggregated in water, is efficiently bound by HSA and all studied cyclodextrins. As noted before, the HSA–STC complex is probably responsible for STC transport in blood [2,11], and the results given here demonstrate that STC can be efficiently extracted from HSA by sugammadex, a clinically used cyclodextrin derivative used for elimination of bio-active alkaloids from blood [15]. 

### 2.3. Molecular Modelling

The above-described experimental results support the formation of STC–cyclodextrin complexes and the obtained binding constants (Table 1) and show a difference between various cyclodextrin ring sizes. This prompted us to perform a molecular modelling study of the proposed complexes to explain these structural features of selectivity in more detail.

As shown earlier (Figure 2), STC overlaps well with rocuronium, whose complex structure with sugammadex is known [20]. 

STC’s low solubility and its strong aggregation in aqueous media [10] hampered potential NMR studies and crystal preparation for an X-ray crystallography study. Thus, to evaluate the structural details of an STC–sugammadex complex, we opted for a molecular modelling approach, quite often used for analyses of CD inclusion complexes [28]. We initially relied on the rocuronium–sugammadex structure (Scheme 1d) obtained by X-ray diffraction (CCDC ref: 172247, [20]).

**Figure 7:**
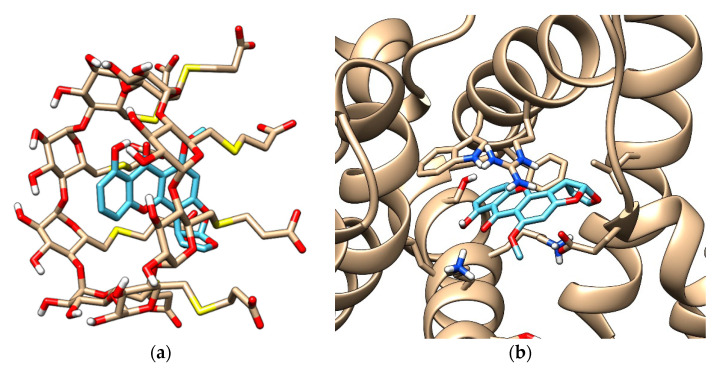
STC (in light blue) in complex with (**a**) sugammadex and (**b**) HSA. (Made in UCSF Chimera 1.16 software package.)

To find the starting conformations for the STC–sugammadex molecular dynamics simulations, STC was manually inserted into the sugammadex molecule instead of rocuronium (Figure 7a). For the STC–HSA complexes, the best STC conformation (*K*_bind_ = −8.7 kcal/mol) was taken (Figure 7b). As can be seen in Figure 7b, STC fits nicely into the HSA binding site I in the vicinity of the Trp214 residue, with which it is able to form π-π interactions. Additionally, hydrogen bonds are also formed between numerous STC oxygen atoms and the nitrogen atoms of Lys195, Lys199, and Arg218, and a hydrophobic interaction is present between the condensed furan rings and Leu238.

Furthermore, the two complexes (STC–sugammadex and STC–HSA complexes) were subjected to 150 ns MD simulations to study their stability and binding constants. In the studied time frame, both complexes were stable (Supp. Info. Figure S2); the slightly higher root-mean-squared deviation (RMSD) of the STC–sugammadex complex can be explained by the fact that STC was manually inserted into the sugammadex cavity, so their complementarity was not as good as in the STC–HSA complex. As can be seen in Table 2, the STC binding constants with sugammadex and HSA seem to be approximately equal (considering the MM/GBSA estimated standard error of 1–3 kcal/mol [29]), but the STC–sugammadex complex has a slightly higher number of intermolecular hydrogen bonds. In these simulations, the entropy term was not calculated, but it was experimentally obtained in the microcalorimetric titration experiments, which showed that the formation of the STC–sugammadex complex is entropically favourable, while formation of the STC–HSA complex is entropically unfavourable. Therefore, the Δ*G*_bind_ of the two complexes differ even more than shown in Table 2, in favour of the STC–sugammadex complex. In conclusion, these results are in accordance with our experimental results, suggesting that sugammadex could also be used as a STC binding molecule to accelerate its elimination from the body.

## 3. Materials and Methods

### 3.1. Reagent Preparation

STC, CD-γ, and HSA were purchased from Sigma Aldrich, Darmstad, Germany; sugammadex (Bridion^®^, Merck Sharp & Dohme Limited, Hertfordshire, UK) was kindly provided by Medika d.d., Zagreb, Croatia; cyclodextrin-β hydrate was obtained from Alfa Aesar, Hengelo, the Netherlands; and sodium cacodylate buffer was prepared by mixing 0.2 M solution of sodium cacodylate (Kefo, Zagreb, Croatia) with 0.2 M hydrochloric acid (Kefo, Zagreb, Croatia) at 25 °C until an *I* of 50 mM and a pH of 7.0 ± 0.02 were reached.

Stock solutions of β-CD (0.005 M), γ-CD (0.01 M), and HSA (1.73 × 10^−4^ M) were prepared in a cacodylate buffer, while in the case of sugammadex, the clinically applied solution (0.05 M) was used. All working solutions for spectrophotometric titrations were prepared in a cacodylate buffer. The STC stock solution (8.9 × 10^−3^ M) was prepared in acetonitrile. For certain experiments, the STC aggregate solution was prepared by dissolving STC (acetonitrile stock solution) in a cacodylate buffer (2.2 × 10^−5^ M) and incubating for 30 min at room temperature. The aggregate formation was confirmed by the unique CD spectrum band at 350 nm according to Jaksic et al., 2019 [10].

For microcalorimetric (ITC) titrations, the original sugammadex solution was diluted by bidistilled water, the STC stock solution was prepared in DMSO (0.015 M), while the STC and HSA working stock solutions were prepared ex tempore in a freshly prepared NaCl solution (Kemika, Zagreb, Croatia) in bidistilled water (*I* = 1.5 mM, pH 7.40 ± 0.02). 

### 3.2. Spectrophotometric Experiments

UV/Vis titrations were performed at room temperature in a quartz cuvette (*l* = 1 cm) by addition of γ-CD (0.001 M), sugammadex (4.6 × 10^−4^ M), β-CD (0.005 M), and HSA (1.74 × 10^−4^ M) to the preformed STC aggregate in a cacodylate buffer (2 × 10^−5^ M). The UV/Vis spectra were recorded on a Varian Cary 100 Bio spectrophotometer (Varian Inc, Palo Alto, CA, USA). 

CD titrations were performed at room temperature in a quartz cuvette (*l* = 1 cm) by addition of γ-CD (0.001 M), sugammadex (4.6 × 10^−4^ M), β-CD (0.005 M), and HSA (1.74 × 10^−4^ M) to the preformed STC aggregate in a cacodylate buffer (2 × 10^−6^ M). The instrument was set to a scanning speed of 200 nm/min, and an average of 3 accumulations of the spectra in the range of 220–550 nm were recorded on a JASCO J-815 spectropolarimeter (Jasco Inc., Tokyo, Japan).

Fluorescent spectra were measured on a Cary Eclipse fluorescence spectrophotometer (Agilent Technologies) in quartz cuvettes (*l* = 1 cm) upon addition of STC (0.5–1 μL) dissolved in acetonitrile (8.9 × 10^−3^ M) to a HSA solution in a cacodylate buffer (2 × 10^−6^ M). Competitive fluorescence experiments were performed in the same cuvette by addition of γ-CD (0.01 M) and β-CD (0.005 M) prepared in a cacodylate buffer and the original sugammadex solution (0.05 M). The excitation wavelength was set to 280 nm, and the emission spectrum was recorded in the 300–500 nm range. The slits were set to 10/10.

All spectra were baseline-corrected in consideration of the volume effect. The data were analysed and visualised using Microsoft Excel (Microsoft Corporation, Redmond, WA, USA) and Origin Lab 9.0 (OriginLab Corporation, Northampton, MA, USA).

### 3.3. Microcalorimetric Titrations

The thermodynamic parameters for STC interactions with HSA and sugammadex were performed at 25 °C on a MicroCal PEAQ-ITC calorimeter (Malvern Panalytical Ltd., UK). The STC stock solution in DMSO (0.015 M) was used in these experiments so that a stable STC aggregate could be prepared in an aqueous solvent with the lowest possible amount of organic solvent (0.067%). Solutions were thoroughly degassed prior to titration under vacuum for 10 min to avoid any bubble formation in the sample cell. The sample cell volume was 200 µL and the burette volume was 40 µL. Titrations were performed by adding aliquots of 1 µL of HSA solution (2.5 × 10^−5^ M) or sugammadex solution (5 × 10^−4^ M) into the calorimeter sample cell containing the pre-formed STC aggregate (10^−5^ M) in a solvent composed of NaCl in bidistilled water (1.5 mM, pH 7.40), which was continuously stirred at 500 rpm. The maximum DMSO concentration in the solvent was 0.067%. Bidistilled water was placed in the reference cell. Differential power (*DP*) was arbitrarily applied to each titration. The interval between successive aliquots was 100 s. To correct for the thermal effect, control experiments were performed in which HSA or sugammadex solutions were added to the solvent, as well as a control experiment in which the solvent was added to the STC solution; all of which were performed under the same conditions as in the original titrations. Thereafter, the heat resulting from dilution was subtracted from the STC–HSA and STC–sugammadex titration data. In competitive titration experiments, all titrations were carried out in the same way as described above, where after the end of the STC (10^−5^ M) titration with HSA (2.5 × 10^−5^ M), the burette was cleaned and loaded with sugammadex (5 ×10^−4^ M), which was then added in small aliquots to the pre-formed STC–HSA complex. The reverse competition experiment was performed similarly; HSA (2.5 × 10^−5^ M) was added to the pre-formed STC–sugammadex complex at the end of the STC (10^−5^ M) titration with sugammadex (5 ×10^−4^ M).

All ITC data were analysed in MicroCal PEAQ-ITC Analysis Software using the One Set Sites Fitting Model.

### 3.4. Molecular Modelling

#### 3.4.1. Docking

Docking studies were performed using AutoDock Vina (The Scripps Research Institute, La Jolla, CA, USA) [30]. Conformation of the HSA molecule was downloaded from RCSB (PDB entry 2BXD) [31] and only the A chain of the crystal structure was used for docking purposes. Missing side chains and hydrogen atoms were added to the protein structure and all ionizable amino acid residues were charged according to their predominant state at pH 7.4. The initial geometry of the ligand molecule (STC) was minimized in HyperChem 8.0 (Hypercube, Inc., Gainesville, FL, USA), and was also charged to represent the most abundant species at pH 7.4, which was calculated at chemicalize.com. The partial charges of STC were set according to Ionescu et al. [32]. Docking was performed in a 22 × 22 × 22 Å grid map, which was centred at the nitrogen NE1 atom in the Trp214 residue (30.017, 76.905, 41.982). In the docking procedure, the receptor molecule was constrained, while all ligand single bonds were allowed to rotate freely. The number of modes and exhaustiveness were set to 100 and the energy range was set to 4 kcal/mol. The lowest energy STC pose was taken for MD simulations.

#### 3.4.2. Molecular Dynamics (MD) Simulations

MD simulations were conducted at 300 K for two different complexes in the presence of water: the STC–sugammadex complex (STC was manually inserted into the cyclodextrin molecule) and the STC–HSA complex (obtained by docking). A GAFF force field was used to model the cyclodextrin and STC molecules, while an AMBER ff14SB force field was used to model HSA, after which the protein molecule was solvated in a truncated octahedral box of TIP3P water molecules spanning a 12 Å thick buffer and was neutralized by Na^+^ ions. After this, the complex was submitted to geometry optimization in the AMBER16 program [33], employing periodic boundary conditions in all directions. In the first 1500 cycles of optimization, the protein and ligand atoms were restrained and only water molecules were optimized, which was followed by 2500 cycles of optimization where all atoms were unrestrained. The systems were then gradually heated from 0 K to 300 K and equilibrated during 30 ps under NVT conditions. Productive and unconstrained MD simulations lasted 150 ns and employed a time step of 2 fs at constant temperature (300 K) and pressure (1 atm). The temperature was held constant using the Langevin thermostat (collision frequency of 1 ps^−1^). The SHAKE algorithm [34] was used to constrain the bonds involving hydrogen atoms and the particle mesh Ewald method [35] was employed to calculate the long-range electrostatic interactions. The non-bonded interactions were truncated at 11.0 Å.

The binding energy, ∆*G*_bind_, of the simulated complexes was calculated using the MM/GBSA (molecular mechanics/generalized born surface area) protocol [29,36], available as a part of AmberTools16 [33]. MM/GBSA is a method for the calculation of ∆*G*_bind_ from snapshots of the MD trajectory [37], with an estimated standard error of 1–3 kcal/mol [37]. ∆*G*_bind_ was calculated in the following manner:

∆*G*_bind_ = <*G*_complex_> − <*G*_protein_> − <*G*_ligand_> (1)

where the symbol < > represents the average value over 500 snapshots collected from the corresponding MD trajectory (every 150th frame). The binding free energies obtained in such a manner were then decomposed to distinguish the contribution of individual amino acid residues to ∆*G*_bind_. This protocol also identifies the nature of the energy change, i.e., the type of interaction, the solvation energy, and the entropic contribution [38,39]. In this case, the entropy term was not calculated.

## 4. Conclusions

Results obtained from three spectrophotometric methods, as well as from a spectrophotometry-independent microcalorimetric method, support the formation of STC–cyclodextrin inclusion complexes. However, STC’s unique ability to strongly aggregate at biologically relevant concentrations in aqueous solutions [10] was a significant challenge in the analysis of the experimental results; thus, in some methods, we studied the binding of STC to various CDs or HSA starting from the STC aggregate, and in some cases we studied interactions of a single STC molecule with the macromolecule host. 

Three methods, CD, UV/Vis spectrophotometry, and ITC microcalorimetry, were performed under conditions in which STC aggregates. The results showed high apparent binding constants between sugammadex and γ-CD, and an almost order of magnitude lower affinity towards β-CD (which is characterised by a smaller ring size than γ-CD derivatives). In parallel, CD, UV/Vis, and ITC data showed that STC binds to HSA (a blood protein known for its role as a transporter of small molecules) with almost a two order of magnitude lower affinity compared to the sugammadex and γ-CD complexes. 

Only the fluorometric experiments, in which intrinsically fluorescent HSA was titrated by addition of an STC acetonitrile solution (in which STC is not aggregated), was able to demonstrate the interaction of HSA and a single STC molecule, revealing the STC micromolar binding constant. This approach, which allowed performing competitive experiments, demonstrated that all cyclodextrins can extract STC from HSA, as proven by recovery of the free HSA fluorescence. These results were confirmed by an ITC competition experiment, also demonstrating the efficient extraction of STC from HSA by sugammadex. Thus, the observed competition mechanism resembles dissociation of the rocuronium–HSA complex caused by rocuronium encapsulation by sugammadex [15,20].

Molecular modelling studies confirmed a good structural fit of STC inside the sugammadex molecule, as well as to the HSA molecule. The binding constants obtained through molecular dynamics simulations showed similar Δ*G*_bind_ for STC–cyclodextrin and STC–HSA complexes, supporting the idea that cyclodextrins could be used for displacement of STC from HSA. This is due to STC’s aromatic and hydrophobic structure which nicely fits inside the hydrophobic sugammadex cavity, which at the same time has six oxygen atoms which can form hydrogen bonds with the hydroxy groups of sugammadex, further stabilizing the STC molecule inside the sugammadex ring. This results in a very stable complex which can even be formed when STC is already bound to HSA.

To conclude, the obtained results can be considered as a proof-of-concept on a molecular level that sugammadex could be used as first aid after an acute intoxication to encapsulate a larger part of STC mycotoxin from serum albumin in blood. These findings strongly support further pharmaco(toxico)kinetic studies with STC and analogous mycotoxins by protocols developed for clinically used rocuronium/sugammadex systems.

## Figures and Tables

**Table 1:** Apparent binding affinities (log*K*_s_) of STC aggregate–host complexes calculated from the data obtained by CD, UV, and ITC titrations. The binding constant of STC to HSA obtained by the change in HSA fluorescence signal fitted to the 1:1 stoichiometry complex.

Complex	log*K*_s_ (CD)	log*K*_s_ (UV)	log*K*_s_ (ITC)	log*K*_s_ (fluo)
STC + CD-β	6.1 ^a^	6.0 ^a^	-	-
STC + CD-γ	6.7 ^a^	6.0 ^b^	-	-
STC + Sugammadex	6.4 ^a^	6.3 ^a^	6.5	-
STC + HSA	4.5 ^a^	5.0 ^c^	6.1 ^e^	6.2 ^d^

^a^ Apparent binding constants are attributed to STC aggregate dissociation caused by STC (a single molecule) binding to a host (cyclodextrin or HSA) and differs from a real binding constant defined by binding of a single STC molecule to a host. ^b^ Significantly increased baseline points toward colloid formation; thus, the titration data can give only an estimation of the binding constant. ^c^ Interference of HSA absorbance and colloidisation allowed for collection of only five titration points, thus allowing only an estimation of the binding constant. ^d^ Titration performed by adding aliquots of STC stock solution in acetonitrile to an aqueous HSA solution. Since STC is not aggregated, this is a real binding constant, defined by binding of a single STC molecule to a host. ^e^ Normalised to one binding site (N = 1).

**Table 2:** Binding statistics (MD) of the tested compounds.

Complex	Number of Intermolecular H Bonds	Δ*G*_bind_ (kcal/mol)
Sagamore–STC	0.48 ± 0.78	−28.90
HSA–STC	0.27 ± 0.59	−30.99

## Data Availability

All data are contained within this manuscript and the Supplementary Materials.

## Supplementary Materials

The following supporting information can be downloaded at: https://www.mdpi.com/article/10.3390/ijms24054485/s1.
