# Supplementary material for: Cyclodextrin-Based Displacement Strategy of Sterigmatocystin from Serum Albumin as a Novel Approach for Acute Poisoning Detoxification
Source: Int J Mol Sci. 2023 Feb 24;24(5):4485. doi: 10.3390/ijms24054485 (PMC10003537; doi:10.3390/ijms24054485)
Supplement: Supplementary file 1 [file ijms-24-04485-s001.zip › ijms-2234428-supplementary.pdf]

Supporting Information

Cyclodextrin-based displacement strategy of sterigmatocystin from serum albumin as a novel approach for acute poisoning detoxification

Daniela Jakšić[1], Maja Šegvić Klarić[1], Hrvoje Rimac[2], Robert Kerep[3] and Ivo Piantanida [4,]*

[1] Department of Microbiology, Faculty of Pharmacy and Biochemistry, University of Zagreb, 10000 Zagreb, Croatia

[2] Department of Medicinal Chemistry, Faculty of Pharmacy and Biochemistry, University of Zagreb, 10000 Zagreb, Croatia

[3] Department of General and Inorganic Chemistry, Faculty of Pharmacy and Biochemistry, University of Zagreb, 10000 Zagreb, Croatia

[4] Division of Organic Chemistry and Biochemistry, Ruđer Bošković Institute, Bijenička Cesta 54, 10000 Zagreb, Croatia

* Correspondence: pianta@irb.hr

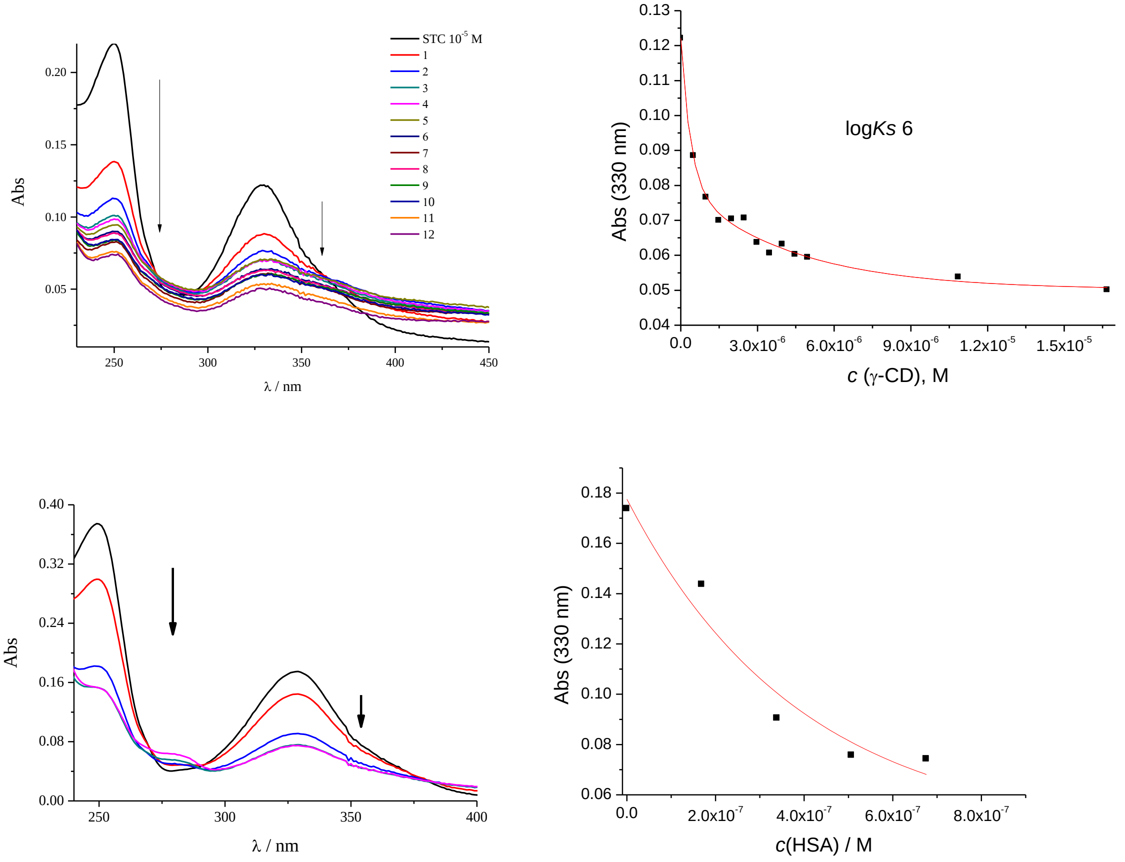

Figure S1. Changes in **STC** UV spectra ($c = 1.8 \times 10^{-5}$ M) upon titration with: cyc-γ (**UP**), HSA (**DOWN**); RIGHT: absorbance changes at λ = 330 nm fitted to the 1:1 stoichiometry complex (—). Performed at pH 7.0, Na cacodylate buffer, $I = 0.05$ M.

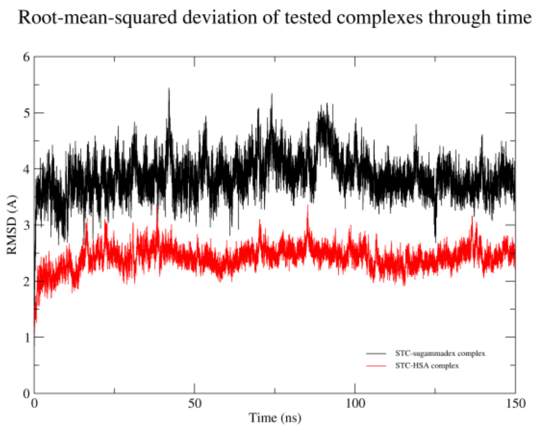

Figure S2. Root-mean-squared deviation of tested complexes through time.

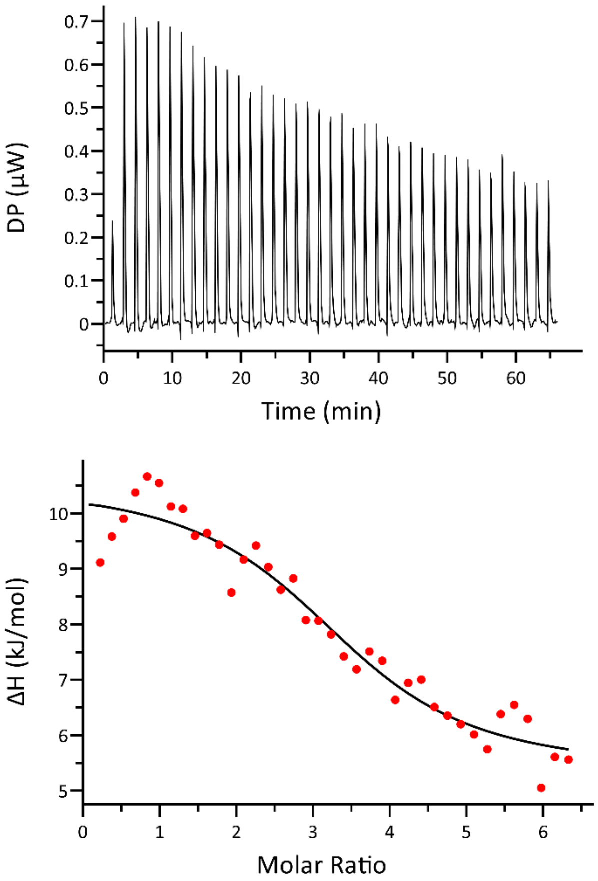

Figure S3. Microcalorimetric titration obtained from HSA (2.5×10^{-5} M) and Sugammadex (5×10^{-4} M in 1.0 µL injections) at 25 °C. Top panel shows the heat in µW per injectant with subtracted baseline. Bottom panel shows the enthalpy change (kJ/mol) as a function of the HSA-Sugammadex molar ratio.
